# Supplementary material for: Density of cannabis outlets vs. cannabis use behaviors and prevalent cannabis use disorder: findings from a nationally-representative survey
Source: PeerJ. 2024 Apr 29;12:e17317. doi: 10.7717/peerj.17317 (PMC11064851; doi:10.7717/peerj.17317)
Supplement: Supplemental Information 2 — Those who refused to answer were excluded from the analysis [file peerj-12-17317-s002.docx]

# **Supplementary Table 1.** Cannabis use status of study participants who reported vs. did not report the number of outlets (answered “don’t know”) within each given distance (row weighted percent ± standard errors, unless otherwise indicated)

#

| **Characteristic** | **Never users** | **Former users** | **Current users** | **P-value** |
| --- | --- | --- | --- | --- |
| **Density of outlets within 400 meters** |  |  |  |  |
| Reported the number of outlets (n=625) | 54.3% ± 1.9% | 17.0% ± 1.5% | 28.6% ± 1.8% | **<0.001** |
| Answered “Don’t know” (n=1121) | 76.5% ± 1.2% | 13.7% ± 1.0% | 9.7% ± 0.9% |  |
| **Density of outlets within 800 meters** |  |  |  |  |
| Reported the number of outlets (n=627) | 53.9% ± 1.9% | 17.8% ± 1.5% | 28.4% ± 1.8% | **<0.001** |
| Answered “Don’t know” (n=1105) | 76.0% ± 1.2% | 13.7% ± 1.0% | 10.3% ± 0.9% |  |
| **Density of outlets within 1200 meters** |  |  |  |  |
| Reported the number of outlets (n=638) | 53.4% ± 1.9% | 20.2% ± 1.6% | 26.5% ± 1.7% | **<0.001** |
| Answered “Don’t know” (n=1105) | 77.1% ± 1.2% | 11.7% ± 0.9% | 11.3% ± 0.9% |  |
| **Density of outlets within 1600 meters** |  |  |  |  |
| Reported the number of outlets (n=643) | 54.1% ± 1.9% | 21.3% ± 1.6% | 24.7% ± 1.7% | **<0.001** |
| Answered “Don’t know” (n=1113) | 76.5% ± 1.2% | 11.3% ± 0.9% | 12.2% ± 1.0% |  |

Those who refused to answer were excluded from the analysis
