# Supplementary material for: Density of cannabis outlets vs. cannabis use behaviors and prevalent cannabis use disorder: findings from a nationally-representative survey
Source: PeerJ. 2024 Apr 29;12:e17317. doi: 10.7717/peerj.17317 (PMC11064851; doi:10.7717/peerj.17317)
Supplement: Supplemental Information 3 [file peerj-12-17317-s003.docx]

# **Supplementary Table 2.** Density of Cannabis Outlets (treating “Don’t Know” as missing values instead of “None”) and Cannabis Use Behaviors

| **Distance and Density** | **Never Users** | **Former Users** | **Current Users** | **Crude OR (95% CI) for Former vs. Never Users** | **Crude OR (95% CI) for Current vs. Never Users** | **Adj. OR (95% CI) for Former vs. Never Users*** | **Adj. OR (95% CI) for Current vs. Never Users*** |
| --- | --- | --- | --- | --- | --- | --- | --- |
| **400 meters** |  |  |  |  |  |  |  |
| None (n=207) | 65.4% ± 3.2% | 15.4% ± 2.5% | 19.3% ± 2.7% | 1 (*Ref.*) | 1 (*Ref.*) | 1 (*Ref.*) | 1 (*Ref.*) |
| 1 outlet (n=188) | 54.4% ± 3.6% | 21.7% ± 3.0% | 23.9% ± 3.1% | **1.70 (1.00, 2.89)** | 1.49 (0.91, 2.45) | **2.14 (1.10, 4.17)** | 1.13 (0.61, 2.08) |
| 2 outlets (n=110) | 41.1% ± 4.7% | 16.3% ± 3.5% | 42.6% ± 4.7% | 1.68 (0.86, 3.29) | **3.52 (2.05, 6.02)** | 1.06 (0.45, 2.50) | 1.79 (0.93, 3.47) |
| 3 outlets (n=120) | 47.5% ± 4.6% | 13.3% ± 3.1% | 39.2% ± 4.5% | 1.19 (0.61, 2.34) | **2.80 (1.67, 4.70)** | 1.14 (0.47, 2.75) | 1.84 (0.94, 3.59) |
| **800 meters** |  |  |  |  |  |  |  |
| None (n=142) | 69.1% ± 3.8% | 14.7% ± 3.0% | 16.2% ± 3.1% | 1 (*Ref.*) | 1 (*Ref.*) | 1 (*Ref.*) | 1 (*Ref.*) |
| 1 outlet (n=196) | 52.2% ± 3.5% | 20.4% ± 2.9% | 27.5% ± 3.2% | **1.83 (1.01, 3.33)** | **2.25 (1.29, 3.95)** | **2.18 (1.06, 4.49)** | 1.88 (0.97, 3.63) |
| 2 outlets (n=110) | 44.7% ± 4.7% | 19.9% ± 3.8% | 35.3% ± 4.6% | **2.09 (1.05, 4.17)** | **3.38 (1.82, 6.26)** | 1.70 (0.71, 4.09) | 1.80 (0.85, 3.85) |
| 3 outlets (n=179) | 49.2% ± 3.7% | 16.1% ± 2.7% | 34.7% ± 3.6% | 1.54 (0.82, 2.90) | **3.01 (1.73, 5.24)** | 1.07 (0.47, 2.44) | 1.66 (0.84, 3.28) |
| **1200 meters** |  |  |  |  |  |  |  |
| None (n=92) | 70.8% ± 4.7% | 15.1% ± 3.7% | 14.1% ± 3.6% | 1 (*Ref.*) | 1 (*Ref.*) | 1 (*Ref.*) | 1 (*Ref.*) |
| 1 outlet (n=225) | 47.1% ± 3.3% | 24.9% ± 2.9% | 28.0% ± 3.0% | **2.47 (1.28, 4.78)** | **2.98 (1.53, 5.84)** | **3.19 (1.46, 6.99)** | **3.14 (1.47, 6.71)** |
| 2 outlets (n=116) | 51.1% ± 4.6% | 18.9% ± 3.6% | 30.0% ± 4.2% | 1.73 (0.81, 3.70) | **2.95 (1.43, 6.10)** | 2.33 (0.90, 6.01) | 2.10 (0.88, 5.04) |
| 3 outlets (n=205) | 53.7% ± 3.5% | 18.0% ± 2.7% | 28.3% ± 3.1% | 1.57 (0.79, 3.12) | **2.65 (1.35, 5.18)** | 1.34 (0.58, 3.11) | 1.80 (0.81, 4.01) |
| **1600 meters** |  |  |  |  |  |  |  |
| None (n=62) | 79.1% ± 5.1% | 11.2% ± 4.0% | 9.6% ± 3.7% | 1 (*Ref.*) | 1 (*Ref.*) | 1 (*Ref.*) | 1 (*Ref.*) |
| 1 outlet (n=216) | 44.0% ± 3.3% | 28.2% ± 3.0% | 27.8% ± 3.0% | **4.51 (1.92, 10.61)** | **5.19 (2.10, 12.83)** | **7.66 (2.79, 21.04)** | **6.66 (2.37, 18.69)** |
| 2 outlets (n=148) | 56.9% ± 4.0% | 16.8% ± 3.1% | 26.2% ± 3.6% | 2.08 (0.84, 5.18) | **3.79 (1.50, 9.56)** | **3.09 (1.02, 9.30)** | **3.63 (1.24, 10.64)** |
| 3 outlets (n=217) | 54.9% ± 3.4% | 20.2% ± 2.7% | 24.9% ± 2.9% | **2.59 (1.09, 6.15)** | **3.72 (1.51, 9.18)** | **3.09 (1.07, 8.89)** | **3.04 (1.08, 8.58)** |

*Adjusted for the participant's sex, age, tobacco smoking status, marital status, income, religion, occupation, educational level, and age of onset of cannabis use
