# Supplementary material for: Density of cannabis outlets vs. cannabis use behaviors and prevalent cannabis use disorder: findings from a nationally-representative survey
Source: PeerJ. 2024 Apr 29;12:e17317. doi: 10.7717/peerj.17317 (PMC11064851; doi:10.7717/peerj.17317)
Supplement: Supplemental Information 4 [file peerj-12-17317-s004.docx]

# **Supplementary Table 3.** Adjusted Beta (95% CI) for cannabis use amount (grams per person per day of use) within the past year by density of cannabis outlets (among current users who smoked the cannabis) without imputation of outlet density or the age of initiation

| **Distance and Density** | **Model 1; Adj. Beta (95% CI)* (n=47)** | **Model 2; Adj. Beta (95% CI)* (n=37)** |
| --- | --- | --- |
| **400 meters** |  |  |
| None (n=28) | 1 (*Ref.*) | 1 (*Ref.*) |
| 1 outlet (n=12) | 2.29 (-1.26, 5.83) | 2.97 (-2.24, 8.18) |
| 2 outlets (n=15) | 4.29 (0.00, 8.58) | 4.84 (-0.49, 10.18) |
| 3 or more outlets (n=16) | 2.56 (-1.08, 6.20) | 3.33 (-1.38, 8.03) |
| **800 meters** |  |  |
| None (n=27) | 1 (*Ref.*) | 1 (*Ref.*) |
| 1 outlet (n=11) | 1.71 (-1.37, 4.80) | 0.88 (-2.50, 4.26) |
| 2 outlets (n=14) | **5.23 (1.32, 9.14)** | **5.09 (1.09, 9.09)** |
| 3 or more outlets (n=20) | 3.01 (-0.23, 6.25) | 2.73 (-0.56, 6.03) |
| **1200 meters** |  |  |
| None (n=28) | 1 (*Ref.*) | 1 (*Ref.*) |
| 1 outlet (n=13) | -1.50 (-3.58, 0.59) | **-2.43 (-4.85, -0.02)** |
| 2 outlets (n=14) | **2.35 (0.56, 4.14)** | 2.21 (0.49, 3.93) |
| 3 or more outlets (n=18) | N/A** | N/A** |
| **1600 meters** |  |  |
| None (n=33) | 1 (*Ref.*) | 1 (*Ref.*) |
| 1 outlet (n=11) | -0.18 (-1.63, 1.28) | -2.06 (-4.00, -0.12) |
| 2 outlets (n=12) | **2.27 (0.56, 3.98)** | **2.46 (0.87, 4.04)** |
| 3 or more outlets (n=19) | N/A** | N/A** |

*Model 1: Adjusted for the participant's sex, age, tobacco smoking status, marital status, income, religion, occupation, educational level, and age of onset of cannabis use (with imputation)

*Model 2: Adjusted for the participant's sex, age, tobacco smoking status, marital status, income, religion, occupation, educational level, and age of onset of cannabis use (without imputation)

**Coefficient not defined because of singularities
