# Supplementary material for: Density of cannabis outlets vs. cannabis use behaviors and prevalent cannabis use disorder: findings from a nationally-representative survey
Source: PeerJ. 2024 Apr 29;12:e17317. doi: 10.7717/peerj.17317 (PMC11064851; doi:10.7717/peerj.17317)
Supplement: Supplemental Information 6 [file peerj-12-17317-s006.pdf]

(ORIGINAL THAI QUESTIONNAIRE)

แบบสอบถาม

การเฝ้าระวังพฤติกรรมการใช้ การเข้าถึงกัญชาและสารเสพติดชนิดอื่นผลกระทบจากการใช้ การรับรู้และความคิดเห็นต่อมาตรการด้านกัญชาในประชากรทั่วไป

หมวด 1. คุณลักษณะของผู้ตอบแบบสอบถาม

A1. เพศกำเนิด [ ] 1.ชาย [ ] 2. หญิง [ ] 3. อื่นๆ [ ] 99. ไม่ตอบ

A3. อายุ ………………..ปี

A4. สถานภาพ [ ] 1. โสด [ ] 2. สมรส [ ] 3. อยู่ด้วยกัน
[ ] 4. หย่าร้าง/เป็นหม้าย [ ] 5. สถานภาพค่อนข้างซับซ้อน [ ] 99. ไม่ตอบ

A5. การศึกษา: กรุณาระบุระดับการศึกษาสูงสุดที่สำเร็จ หรือคุณวุฒิระดับสูงสุดที่ได้รับ
[ ] 1. น้อยกว่ามัธยมศึกษาตอนปลาย [ ] 2. มัธยมศึกษาตอนปลายหรือเทียบเท่า
[ ] 3. ปวช. / ประกาศนียบัตรวิชาชีพ [ ] 4. ปวส. / ประกาศนียบัตรวิชาชีพชั้นสูง/อนุปริญญา
[ ] 5. เรียนมหาวิทยาลัยแต่ไม่จบปริญญา (หากผู้ตอบเป็นนักศึกษา ป.ตรี กรุณาตอบข้อนี้)
[ ] 6. ปริญญาตรี [ ] 7. ปริญญาโท [ ] 8. ปริญญาเอก [ ] 99. ไม่ตอบ

A6. อาชีพปัจจุบันที่มีรายได้หลัก
[ ] 1. ข้าราชการ/ รัฐวิสาหกิจ [ ] 2. พนักงานบริษัทเอกชน [ ] 3. ค้าขายรายย่อย/ บริการรายย่อย
[ ] 4. ธุรกิจส่วนตัว/ ผู้ประกอบการ [ ] 5. ผู้ใช้แรงงาน/ รับจ้างทั่วไป [ ] 6. เกษียณอายุ/ พ่อบ้าน/แม่บ้าน
[ ] 7. เกษตรกร/ ประมง [ ] 8. วิชาชีพอิสระ อาทิ ทนายความ สถาปนิก
[ ] 9. อาชีพอิสระ อาทิ ไรเดอร์ ขายของออนไลน์ [ ] 10. นักเรียน/ นักศึกษา [ ] 11. ว่างงาน
[ ] 12. อื่นๆ ระบุ ………………………………….. [ ] 99. ไม่ตอบ

A7. รายได้ส่วนบุคคลต่อเดือน: ในช่วง 12 เดือนที่ผ่านมา คุณมีรายได้ เดือนละประมาณเท่าไร
[1] น้อยกว่า 5,000 บาท [2] 5,000 - 9,999 บาท [3] 10,000 - 14,999 บาท
[4] 15,000 - 19,999 บาท [5] 20,000 - 24,999 บาท [6] 25,000 - 29,999 บาท
[7] 30,000 - 34,999 บาท [8] 35,000 - 39,999 บาท [9] 40,000 - 44,999 บาท
[10] 45,000 - 49,999 บาท [11] 50,000 บาทหรือมากกว่า [99] ไม่ตอบ

A9. ศาสนา [ ] 1. อิสลาม [ ] 2. พุทธ [ ] 3. คริสต์ [ ] 4. อื่นๆ ………………. [ ] 99. ไม่ตอบ

หมวด 2. แบบแผนพฤติกรรมการใช้กัญชา

"การใช้กัญชาในที่นี้ หมายถึง การให้ร่างกายได้รับสารในพืชกัญชาด้วยวิธีใดก็ได้ ไม่ว่าจะเพื่อการแพทย์หรือเพื่อความเพลิดเพลิน อาทิเช่น ดูดปุ๊น (มวนสูบ) ผสมบุหรี่สูบ ใส่ไปป์หรือบ้องแห้งแล้วสูบ ใส่บ้องไอน้ำแล้วสูบ กินหรือดื่ม หยดใต้ลิ้น เหน็บ/สวนทวาร บรรจุในแคปซูลและกิน พ่น/ทา/อาบน้ำภายนอก ใส่ในบุหรี่ไฟฟ้า และหมาย

รวมถึงผลิตภัณฑ์ผสมกัญชาต่างๆ ด้วย อาทิ ขนมขบเคี้ยว ลูกอม เครื่องดื่ม อาหารปรุงสำเร็จ ฯลฯ (มีสาร THC ไม่เกิน 0.2%) "

<u>การใช้กัญชา</u>

B1_1. ในชีวิตนี้ คุณเคยใช้กัญชาหรือไม่?

[ ] 0. ไม่เคย (ข้ามไปข้อ B2_1) [ ] 1. เคย [ ] 99. ไม่ตอบ (ข้ามไปข้อ B2_1)

B1_3. คุณเริ่มใช้กัญชาครั้งแรกเมื่ออายุ……………….ปี (หากไม่แน่ใจ ใส่เลข 88 หากไม่ตอบ ใส่เลข 99)

B1_4. <u>วัตถุประสงค์หลัก</u>ในการใช้กัญชาของคุณในช่วงชีวิตนี้ตรงกับข้อใดที่สุด **(ตอบเพียงข้อเดียว)**

[ ] 1. สันทนาการ (เพื่อความเพลิดเพลิน-ผ่อนคลาย เข้าสังคม)

[ ] 2. เหตุผลทางการแพทย์/รักษาโรค (เช่น แก้ปวดเมื่อย โรคมะเร็ง โรคลมชัก)

[ ] 3. ช่วยให้นอนหลับ [ ] 4. ช่วยให้เจริญอาหาร [ ] 5. อื่นๆ ระบุ …………………… [ ] 99. ไม่ตอบ

B1_8. คุณใช้กัญชาโดยวิธีใดบ่อยที่สุด **<u>(ตอบได้เพียงข้อเดียว)</u>**

[ ] 1. ดูดปุ๊น (มวนสูบ) [ ] 2. ผสมบุหรี่สูบ [ ] 3. ใส่ไปป์ หรือบ้องแห้งแล้วสูบ

[ ] 4. ใส่บ้องไอน้ำ แล้วสูบ [ ] 5. กินหรือดื่ม [ ] 6. หยดใต้ลิ้น

[ ] 7. เหน็บ/สวนทวาร [ ] 8. บรรจุในแคปซูลและกิน [ ] 9. พ่น/ทา/อาบน้ำภายนอก

[ ] 10. ใส่ในบุหรี่ไฟฟ้า (vaporization) [ ] 11. อื่นๆ ระบุ ………………..[ ] 99. ไม่ตอบ

<u>แบบคัดกรองอาการติดกัญชา (CUDIT-R Questionnaire)</u>

B3_1. <u>ในช่วง 12 เดือนที่ผ่านมา</u> คุณใช้กัญชาบ่อยแค่ไหน

[0] ไม่ได้ใช้เลย **(ข้ามไปข้อ B4_1)**

[1] 1-3 ครั้งต่อปี [2] 4-7 ครั้งต่อปี [3] 8-11 ครั้งต่อปี [4] เดือนละครั้ง

[5] เดือนละ 2-4 ครั้ง [6] สัปดาห์ละ 2-3 ครั้ง [7] สัปดาห์ละ 4-6 ครั้ง [8] สัปดาห์ละ 7 ครั้ง (ทุกวัน) [9] ไม่ตอบ

B3_2. ในวันที่คุณใช้กัญชา คุณเมากัญชาหรือ "ลอย" ประมาณกี่ชั่วโมง

[0] น้อยกว่า 1 ชั่วโมง [1] 1 หรือ 2 ชั่วโมง [2] 3 หรือ 4 ชั่วโมง

[3] 5 หรือ 6 ชั่วโมง [4] 7 ชั่วโมงหรือมากกว่า [9] ไม่ตอบ [ ] ไม่มีอาการลอย

B3_3. ในช่วง 6 เดือนที่ผ่านมา คุณพบว่าตนเองเมื่อเริ่มใช้กัญชาแล้วหยุดไม่ได้ บ่อยแค่ไหน

[ ] 0. ไม่เคย [ ] 1. น้อยกว่าเดือนละครั้ง [ ] 2. เดือนละครั้ง

[ ] 3. สัปดาห์ละครั้ง [ ] 4. ทุกวัน หรือเกือบทุกวัน [ ] 9. ไม่ตอบ

B3_4. ในช่วง 6 เดือนที่ผ่านมา คุณไม่สามารถทำสิ่งต่างๆ ได้เท่าที่ควร เพราะการใช้กัญชา บ่อยแค่ไหน?

[ ] 0. ไม่เคย [ ] 1. น้อยกว่าเดือนละครั้ง [ ] 2. เดือนละครั้ง

[ ] 3. สัปดาห์ละครั้ง [ ] 4. ทุกวัน หรือเกือบทุกวัน [ ] 9. ไม่ตอบ

B3_5. ในช่วง 6 เดือนที่ผ่านมา คุณเสียเวลามากไปกับการหา ใช้ และถอนฤทธิ์กัญชา บ่อยแค่ไหน?

[ ] 0. ไม่เคย [ ] 1. น้อยกว่าเดือนละครั้ง [ ] 2. เดือนละครั้ง

[ ] 3. สัปดาห์ละครั้ง [ ] 4. ทุกวัน หรือเกือบทุกวัน [ ] 9. ไม่ตอบ

B3_6. ในช่วง 6 เดือนที่ผ่านมา คุณประสบปัญหาเรื่องความจำหรือการตั้งสมาธิ หลังจากใช้กัญชา บ่อยแค่ไหน?

[ ] 0. ไม่เคย [ ] 1. น้อยกว่าเดือนละครั้ง [ ] 2. เดือนละครั้ง

[ ] 3. สัปดาห์ละครั้ง [ ] 4. ทุกวัน หรือเกือบทุกวัน [ ] 9. ไม่ตอบ

B3_7. คุณใช้กัญชาในเวลาที่อาจเกิดอันตรายได้ เช่น การขับรถ ควบคุมเครื่องจักร หรือดูแลเด็ก บ่อยแค่ไหน?

[ ] 0. ไม่เคย [ ] 1. น้อยกว่าเดือนละครั้ง [ ] 2. เดือนละครั้ง

[ ] 3. สัปดาห์ละครั้ง [ ] 4. ทุกวัน หรือเกือบทุกวัน [ ] 9. ไม่ตอบ

B3_8. คุณเคยคิดจะลดหรือเลิกใช้กัญชาหรือไม่?

[ ] 0. ไม่เคย [ ] 2. เคยคิด แต่ไม่ได้คิดในช่วง 6 เดือนที่ผ่านมานี้

[ ] 4. เคยคิดในช่วง 6 เดือนที่ผ่านมานี้ [ ] 9. ไม่ตอบ

(รวมคะแนนจาก B3_1 – B3_8 = ... คะแนน (โดยไม่นับโค้ด 9 ไม่ตอบ) **หากได้ 12 คะแนนขึ้นไป กรุณาแจ้งผู้เก็บข้อมูลดังต่อไปนี้)**

"คุณได้คะแนนการคัดกรองอาการติดกัญชา 12 คะแนนหรือมากกว่า ซึ่งเข้าข่ายมีความเสี่ยงต่ออาการติดกัญชา ขอแนะนำให้ปรึกษาแพทย์ตามรายละเอียดเบื้องต้นในแผ่นพับนี้ เพื่อตรวจโดยละเอียดอีกครั้งหนึ่ง" (จากนั้นให้ผู้เก็บข้อมูลมอบแผ่นผับให้อาสาสมัคร)

<u>การสูบบุหรี่ (ไม่รวมบุหรี่ไฟฟ้า)</u>

B6_1. ตลอดชีวิตที่ผ่านมา คุณเคยสูบบุหรี่หรือยาเส้น รวมมากกว่า 5 ซอง หรือ 100 มวนหรือไม่

[ ] 1. ไม่เคยสูบบุหรี่เลย (ข้ามไปตอบข้อ C1_1) [ ] 2. เคยสูบ แต่รวมไม่เกิน 5 ซอง หรือ 100 มวน

[ ] 3. เคย

B6_2. คุณสูบบุหรี่ครั้งล่าสุดเมื่อไร

[ ] 1. นานกว่า 12 เดือน [ ] 2. นานกว่า 30 วันที่ผ่านมา แต่ภายใน 12 เดือน

[ ] 3. นานกว่า 1 สัปดาห์ที่ผ่านมา แต่ภายใน 30 วัน [ ] 4. ภายใน 1 สัปดาห์ที่ผ่านมา

หมวด 3. การเข้าถึงและการพบเห็นโฆษณากัญชา การได้รับควันกัญชามือสอง ความคิดเห็นด้านนโยบายจัดการกัญชา ทัศนคติและบรรทัดฐานเรื่องการใช้กัญชา

C1_6. เท่าที่คุณทราบ ในระยะ 400 เมตร รอบบ้านคุณ (เดินประมาณ 5 นาที) มีจุดปลูกและจุดขายกัญชารวมทั้งสิ้น

ประมาณกี่จุด.........................จุด (ถ้าไม่แน่ใจ กรอก 88 ถ้าไม่ต้องการตอบ กรอก 99)

C1_7. เท่าที่คุณทราบ ในระยะ 800 เมตร รอบบ้านคุณ (เดินประมาณ 10 นาที) มีจุดปลูกและจุดขายกัญชา รวมทั้งสิ้นประมาณกี่จุด.........................จุด (ถ้าไม่แน่ใจ กรอก 88 ถ้าไม่ต้องการตอบ กรอก 99)

C1_8. เท่าที่คุณทราบ ในระยะ 1,200 เมตร รอบบ้านคุณ (เดินประมาณ 15 นาที) มีจุดปลูกและจุดขายกัญชา รวมทั้งสิ้นประมาณกี่จุด......................... จุด (ถ้าไม่แน่ใจ กรอก 88 ถ้าไม่ต้องการตอบ กรอก 99)

C1_9. เท่าที่คุณทราบ ในระยะ 1,600 เมตร รอบบ้านคุณ (เดินประมาณ 20 นาที) มีจุดปลูกและจุดขายกัญชา รวมทั้งสิ้นประมาณกี่จุด.........................จุด (ถ้าไม่แน่ใจ กรอก 88 ถ้าไม่ต้องการตอบ กรอก 99)

TRANSLATION
Questionnaire
Surveys on cannabis use, access, effects, awareness, and opinions towards cannabis-related measures in the general population

Section 1. Characteristics of the Study Participants

A1. Gender assigned at birth
[ ] 1. Male [ ] 2. Female [ ] 3. Others [ ] 99. Refuse to answer

A3. Age (text field)

A4. Marital status [ ] 1. Single [ ] 2. Married [ ] 3. Co-habitation [ ] 4. Widowed/Divorced [ ] 5. It's complicated [ ] 99. Refuse to answer

A5. Education: Please specify the highest level of education completed or highest qualitifcation earned
[ ] 1. Less than high school
[ ] 2. High school
[ ] 3. Vocational certificate
[ ] 4. Associate's degree
[ ] 5. Studied at university but did not graduate (check this box if you're currently an undergraduate student)
[ ] 6. Bachelor's degree
[ ] 7. Master's degree
[ ] 8. Doctorate
[ ] 99. Refuse to answer

A6. Occupation that is your primary source of income
[ ] 1. Civil servant / state enterprise
[ ] 2. Corporate employee
[ ] 3. Small-scale vendor / service provider
[ ] 4. Business owner / enterpreneur
[ ] 5. Manual labor / general worker
[ ] 6. Retired / homemaker
[ ] 7. Agriculture / fishery
[ ] 8. Independent professionals, e.g., lawyer, architect
[ ] 9. Independent occupations, e.g., delivery riders, online shops
[ ] 10. Student
[ ] 11. Unemployed
[ ] 12. Others
[ ] 99. Refuse to answer

A7. Personal Monthly Income: In the past 12 months, how much do you typically earn per month?
[1] No more than 5,000 THB
[2] 5,001 to 9,999 THB
[3] 10,000 to 14,999 THB
[4] 15,000 to 19,999 THB
[5] 20,000 to 24,999 THB
[6] 25,000 to 29,999 THB
[7] 30,000 to 34,999 THB
[8] 35,000 to 39,999 THB
[9] 40,000 to 44,999 THB
[10] 45,000 to 49,999 THB
[11] 50,000 THB or more
[99] Refuse to answer

A9. Religion
[ ] 1. Islam
[ ] 2. Buddhism
[ ] 3. Christianity
[ ] 4. Others
[ ] 99. Refuse to answer

Section 2. Patterns of Cannabis Use Behaviors
"Cannabis use" refers to the intake of cannabis in any form to the body, whether for medical or recreational purposes. This include rolling in a joint, mixing with cigarette, using a pipe or dry bong, using a water bong, eating or drinking, sublingual drops, suppository, ingestion of capsules, spraying / applying / bathing externally, use in electronic cigarette, as well as the use of other cannabis-infused products including snacks, candy drops, drinks, food, etc. (THC content of or more than 0.2%)

Cannabis Use
B1_1. In this lifetime, have you ever used cannabis?
[ ] 0. Never (skip to B2_1)
[ ] 1. Yes
[ ] 9. Refuse to answer (skip to B2_1)

B1_3. Age in which you first used cannabis (if not sure, add 888; if refuse to answer, add 999). [ ] [ ] [ ]

B1_4. What is the main purpose of your cannabis use in your lifetime? (only one answer allowed)
[ ] 1. Recreation (for enjoyment-relaxation, socialization)
[ ] 2. Medical reason / treatment (e.g., for fatigue, cancer, seizure)
[ ] 3. As a sleep-aid
[ ] 4. To increase appetite
[ ] 5. Others
[ ] 99. Refuse to answer

B1_8. What is your most common method of cannabis use? (only one answer allowed)
[ ] 1. Rolling in a joint
[ ] 2. Mixing with cigarette
[ ] 3. Using a pipe or dry bong
[ ] 4. Using a water bong
[ ] 5. Eating or drinking
[ ] 6. Sublingual drops
[ ] 7. Suppository
[ ] 8. Ingestion of capsules
[ ] 9. Spraying / applying / bathing externally
[ ] 10. Use in electronic cigarette (voporization)
[ ] 11. Others
[ ] 99. Refuse to answer

The Cannabis Use Disorder Identification Test - Revised (CUDIT-R) Questionnaire
B3_1. In the past 12 months, how often do you use cannabis?
[0]. Never (skip to B4_1)
[1]. 1-3 times per year
[2]. 4-7 times per year
[3]. 8-11 times per year
[4]. Once a month
[5]. 2-4 times per month
[6]. 2-3 times per week
[7]. 4-6 times per week
[8]. 7 times per week (daily)

[9]. Refuse to answer

B3_2. How many hours were you high or "floating" on a typical day when you had been using cannabis?
[0]. Less than 1 hour
[1]. 1 or 2 hours
[2]. 3 or 4 hours
[3]. 5 or 6 hours
[4]. 7 hours or more
[9]. Refuse to answer
[10]. I don't get high

B3_3. How often during the past 6 months did you find that you were not able to stop using cannabis once you had started?
[ ] 0. Never
[ ] 1. Less than once a month
[ ] 2. Once a month
[ ] 3. Once a week
[ ] 4. Daily or almost daily
[ ] 9. Refuse to answer

B3_4. How often during the past 6 months did you fail to do what was normally expected from you because of using cannabis?
[ ] 0. Never
[ ] 1. Less than once a month
[ ] 2. Once a month
[ ] 3. Once a week
[ ] 4. Daily or almost daily
[ ] 9. Refuse to answer

B3_5. How often in the past 6 months have you devoted a great deal of your time to getting, using, or recovering from cannabis?
[ ] 0. Never
[ ] 1. Less than once a month
[ ] 2. Once a month
[ ] 3. Once a week
[ ] 4. Daily or almost daily
[ ] 9. Refuse to answer

B3_6. How often in the past 6 months have you had a problem with your memory or concentration after using cannabis?
[ ] 0. Never
[ ] 1. Less than once a month
[ ] 2. Once a month
[ ] 3. Once a week
[ ] 4. Daily or almost daily
[ ] 9. Refuse to answer

B3_7. How often do you use cannabis in situations that could be physically hazardous, such as driving, operating machinery, or caring for children:
[ ] 0. Never
[ ] 1. Less than once a month
[ ] 2. Once a month
[ ] 3. Once a week
[ ] 4. Daily or almost daily
[ ] 9. Refuse to answer

B3_8. Have you ever thought about cutting down, or stopping, your use of cannabis?
[ ] 0. Never
[ ] 2. Yes, but not within the past 6 months
[ ] 4. Yes, in the past 6 months
[ ] 9. Refuse to answer

Tally the points from B3_1 to B3_8 = ... points (not including the "9. Refuse to answer"). If the total is 12 points or higher, please inform the participant as follows)<br>"Your cannabis acction screening score was at 12 points or higher, which puts you at risk of cannabis use disorder. Please consult a healthcare professional according to information in this brochure to be examined in greater detail" (Data collection present the participant with the brochure)

Smoking (not including electronic cigarettes)
B6_1. In your lifetime, have you smoked more than 5 packs or 100 sticks of cigarettes or loose-leaf tobacco?
[ ] 1. I never smoked
[ ] 2. I smoked, but fewer than 5 packs or 100 sticks in total
[ ] 3. Yes

B6_2. When was the last time you smoked?
[ ] 1. More than 12 months ago
[ ] 2. More than 30 days ago but within the past 12 months
[ ] 3. More than 1 week ago but within the past 30 daysw
[ ] 4. Within the past 1 week

Section 3. Access and exposure to cannabis advertisement, exposure to secondhand cannabis smoke, opinions regarding cannabis control policy, attitude and norms regarding cannabis use
C1_6. As you are aware, within 400 meters from your home (approximately 5 minutes' walk), how many cannabis growing and selling points are there? .... points (if not sure, write 88; if refuse to answer, write 99).

C1_7. As you are aware, within 800 meters from your home (approximately 5 minutes' walk), how many cannabis growing and selling points are there? .... points (if not sure, write 88; if refuse to answer, write 99).

C1_8. As you are aware, within 1200 meters from your home (approximately 5 minutes' walk), how many cannabis growing and selling points are there? .... points (if not sure, write 88; if refuse to answer, write 99).

C1_9. As you are aware, within 1600 meters from your home (approximately 5 minutes' walk), how many cannabis growing and selling points are there? .... points (if not sure, write 88; if refuse to answer, write 99).
